# Visuo-Motor Affective Interplay: Bonding Scenes Promote Implicit Motor Pre-dispositions Associated With Social Grooming–A Pilot Study

**DOI:** 10.3389/fpsyg.2022.817699

**Published:** 2022-04-07

**Authors:** Olga Grichtchouk, Jose M. Oliveira, Rafaela R. Campagnoli, Camila Franklin, Monica F. Correa, Mirtes G. Pereira, Claudia D. Vargas, Isabel A. David, Gabriela G. L. Souza, Sonia Gleiser, Andreas Keil, Vanessa Rocha-Rego, Eliane Volchan

**Affiliations:** ^1^Instituto de Biofísica Carlos Chagas Filho, Avenida Carlos Chagas Filho, Centro de Ciências da Saúde, Universidade Federal do Rio de Janeiro, Rio de Janeiro, Brazil; ^2^Instituto Biomédico, Universidade Federal Fluminense, Niterói, Brazil; ^3^Instituto de Biologia, Universidade Federal Fluminense, Niterói, Brazil; ^4^Instituto de Psiquiatria, Universidade Federal do Rio de Janeiro, Rio de Janeiro, Brazil; ^5^Departamento de Ciências Biológicas, Universidade Federal de Ouro Preto, Ouro Preto, Brazil; ^6^Department of Psychology, Center for the Study of Emotion and Attention, University of Florida, Gainesville, FL, United States

**Keywords:** social touch, bonding scenes, EMG, fingers flexor muscle, social grooming, dyads

## Abstract

Proximity and interpersonal contact are prominent components of social connection. Giving affective touch to others is fundamental for human bonding. This brief report presents preliminary results from a pilot study. It explores if exposure to bonding scenes impacts the activity of specific muscles related to physical interaction. Fingers flexion is a very important component when performing most actions of affectionate contact. We explored the visuo-motor affective interplay by priming participants with bonding scenes and assessing the electromyographic activity of the fingers flexor muscle, in the absence of any overt movements. Photographs of dyads in social interaction and of the same dyads not interacting were employed. We examined the effects upon the electromyographical activity: (i) during the passive exposure to pictures, and (ii) during picture offset and when expecting the signal to perform a fingers flexion task. Interacting dyads compared to matched non-interacting dyads increased electromyographic activity of the fingers flexor muscle in both contexts. Specific capture of visual bonding cues at the level of visual cortex had been described in the literature. Here we showed that the neural processing of visual bonding cues reaches the fingers flexor muscle. Besides, previous visualization of bonding cues enhanced background electromyographic activity during motor preparation to perform the fingers flexion task, which might reflect a sustained leakage of central motor activity downstream leading to increase in firing of the respective motor neurons. These data suggest, at the effector level, an implicit visuo-motor connection in which social interaction cues evoke intrinsic dispositions toward affectionate social behavior.

## Introduction

Given their primordial relevance for survival, it is adaptive for an observer to be constantly attuned to cues indicating social bonding. Furthermore, the identification of these cues is expected to promptly promote actions (or pre-dispositions to act) in order to increase proximity and social physical contact. The visuo-motor interplay between visual bonding cues and pre-dispositions to give social touch, is still underexplored at the physiological level. The present brief report aims to contribute to this issue in a preliminary sample of eight observers.

Previous studies in children and non-human primates have demonstrated the salience of visual bonding cues and their ability to trigger prosocial behaviors: Over and Carpenter (2009) observed that exposure to visual social interaction cues heightened children’s tendency to display helping behavior. Berthier and Semple (2018), studying semi-free-ranging Barbary monkeys, showed that when bystanders observed a pair of conspecifics involved in social grooming, they rapidly engaged in the search for a partner and initiated grooming as the groomer. Dunbar (2010) described that giving social grooming is mostly performed by hand and fingers movements and that allo-grooming (grooming of others) has high relevance for bonding both in human and non-human primates.

Physiologically, human visual perception has been shown to be highly sensitive to social stimuli conveying information regarding dyadic interaction (Abassi and Papeo, 2020). Papeo (2020) remarked “… the facing dyad in the visual cortex may be the earliest rudimentary representation of social interaction.” Indeed, the study of Over and Carpenter (2009) mentioned above used facing dyads as visual stimuli, indicating that the sensitivity to facing vs. non-facing dyads as social interaction cues is present very early in life.

In our previous electrophysiological investigation (Campagnoli et al., 2015), we employed dyadic stimuli as bonding visual cues to test the hypothesis that viewing dyadic interactions facilitates overt motor action consistent with grooming. The dyadic stimuli consisted of “real world” pictures portraying two children or a child and an adult, either interacting or non-interacting. After looking at each picture for a few seconds, participants performed a paced flexion of fingers on a very soft cloth. In the readiness potential, an electroencephalographic marker of motor preparation, a facilitatory effect was visible when comparing pictures of interacting dyads to non-interacting ones. This facilitatory effect suggested the recruitment of pre-set cortical motor repertoires related to caress- or grooming-like movements, consistent with a pre-set integration between visual and overt motor processing underlying intrinsic motivations for affectionate attitudes toward others, at the level of cortical function.

Building on these previous findings, the present study set out to examine the extent to which the presentation of social bonding scenes prompts heightened background, or covert, activity in the effector system, specifically the fingers flexor muscle, in the absence of any overt movements. To enhance the compatibility of bonding stimuli with pre-dispositions for social grooming, the paradigm included a task of fingers flexion over a soft cloth. A newly elaborated catalog was used (Silva et al., 2017). Each selected dyad (two children or a child and an adult) was photographed against the same background in an interacting attitude and in a non-interacting one, providing the matching control. This allowed a paired picture-level comparison, not available in Campagnoli et al. (2015) study.

The aim of the present study is to examine the effects of capturing bonding visual cues upon the electromyographical activity of the finger flexor. We analyze these effects in two different and distinct contexts: (i) during the passive exposure to bonding pictures, and (ii) when expecting the signal to perform a finger flexion task (motor preparation period). We expected an increase in electromyographic activity when viewing interacting dyads compared to matched non-interacting dyads. Besides, we hypothesized that the previous visualization of these bonding cues would enhance background electromyographic activity in preparation for the finger flexion task. This finding would lend physiological support, at the effector level, to our overarching hypothesis that social visual cues alter behavioral dispositions, facilitating social grooming behavior.

## Materials and Methods

### Participants

Eleven right-handed university students participated in the study. The Ethics Review Board of the Institute of Psychiatry of the Federal University of Rio de Janeiro approved this study, and the participants provided written informed consent before assessment. The study has been conducted in accordance with the Declaration of Helsinki. Participants were blind to the purpose of the experiment and were informed that they could withdraw from the experiment at any time. Data was collected before the COVID-19 outbreak.

Due to problems in signal acquisition or excessive signal noise, three participants were excluded. Therefore, the final sample comprised eight participants (mean age: 23.6 5.55 y.o., 7 women).

### Visual Stimuli

The stimuli were selected from a previously created catalog of photographs designed for research purposes (Silva et al., 2017). All pictures portrayed two individuals. The dyads always included a baby or child, with the other individual being another baby/child or a parent-like adult. Pictures of babies/children were employed to enhance the implicit pre-dispositions to grooming as proposed by Brosch et al. (2007). Each dyad represented two different conditions: bonding and control. For each dyad, the two conditions were photographed in the same environment, which could be indoors or outdoors (school, garden, park, playground). We established the following criteria for choosing the matched pair. For the bonding condition, (i) there should be body contact between individuals, and (ii) they should be gazing at each other, unless they were in a close embrace which precluded gazing. For the control condition, (i) there should be no body contact between the dyad, and (ii) at least one of the individuals should be holding/grabbing an object. As the goal of this study relies on capture of social interaction from the scenes and some pictures of bonding dyads portray hand-touching, the later criterion aimed to exclude purely mimicry of hands in flexion postures.

**FIGURE 1:**
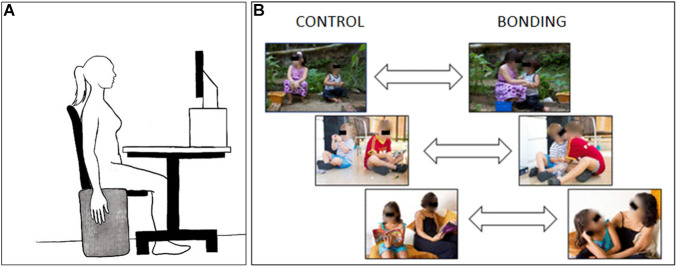
Schematic diagram of the experimental setup. **(A)** Participant‘s positioning during the experimental session. **(B)** Three examples of visual stimuli pairs. Pictures at the right side depict the interacting bonding dyads and those at the left side, their respective matched non-interacting controls. In the experimental session the eyes in the pictures were not covered.

The selected pairs of pictures (*N* = 26) (see three examples in Figure 1B) were edited so that each dyad appeared in the center of the picture. All pictures had the same height; width was adjusted by adding two gray borders at the lateral edges when necessary. The pictures were standardized to 1024 × 768 pixels. Analysis of physical properties revealed that the bonding and control pictures were matched in terms of brightness [t(50) = 0.10, *p* = 0.92], contrast [t(50) = −0.64, *p* = 0.52], and spatial frequency [t(50) = 0.56, *p* = 0.58].

The pictures were analyzed for the quantification of hands appearing in flexed postures in the bonding (e.g., touching the partner) and control conditions (e.g., holding objects). Pictures belonging to the control condition had more flexed hands, on average, than those of the bonding condition (Control: 3.3 ±0.84; Bonding: 2.6 ±0.86). Therefore, if motor mimicry of hands in flexion postures, rather than the global perception of social interaction, primarily influenced the EMG activity, the control condition should evoke higher EMG activity than the bonding condition.

The 26 pairs of bonding and control pictures were further subdivided in two sets (set 1 and set 2) each with 13 pairs. Thus, each set was composed of 13 bonding pictures and 13 control pictures. The bonding and control pictures of each set were presented in blocks (control block and bonding block). Each block was formed by the 13 pictures, always presented in the first part of the block, followed by the repetition of 7 of these pictures, for a total of 20 pictures per block. The repeated pictures aimed to increase the number of stimuli per block. The sequence of the first 13 pictures was randomized each time the block was presented. The sequence of the seven duplicated ones was also randomized.

### Data Collection

Two computers controlled the picture presentation and data acquisition of the physiological signals, running, respectively, E-Prime^®^ software version 2.0 Professional (Psychology Software Tools Inc., Pittsburgh, PA, United States) to present the stimuli on the monitor screen and sync signal registration, and Acknowledge (BIOPAC Systems Inc., Goleta, CA, United States) software to record the physiological signals.

### Electrophysiological Recording

The electromyographic (EMG) signal of the activity of the *flexor digitorum superficialis* muscle of the right arm was recorded through a pair of surface electrodes placed 2 centimeters apart (Ag-AgCl) and a reference electrode was fixed to the left lateral epicondyle. A Biopac MP150 system, set with a gain of 1000, a band-pass filter from 20 to 500 Hz and a sample rate of 1,000 Hz was employed for recording. Synchronizing pulses (triggers) sent from the stimuli-presentation computer were recorded along with the signals.

### Procedure

The participant was seated in front of a monitor screen (15-inch) with the head on a forehead and chin support, and the EMG electrodes were attached to the right forearm. The distance from the screen was 44 cm and the stimuli at the screen had 20 degrees (width) and 15 degrees (height) of visual angle. At the right side of the chair, in an upright position, there was an apparatus consisting of a thin rectangular piece of wood covered with a very soft cloth. During the recordings, the participant was asked to keep the right arm relaxed at their side, aiming at reducing artifacts and enabling the recording of low EMG amplitudes (Figure 1A). A training session familiarized the participant with the experiment dynamics and served for electromyography signal checking.

**FIGURE 2:**
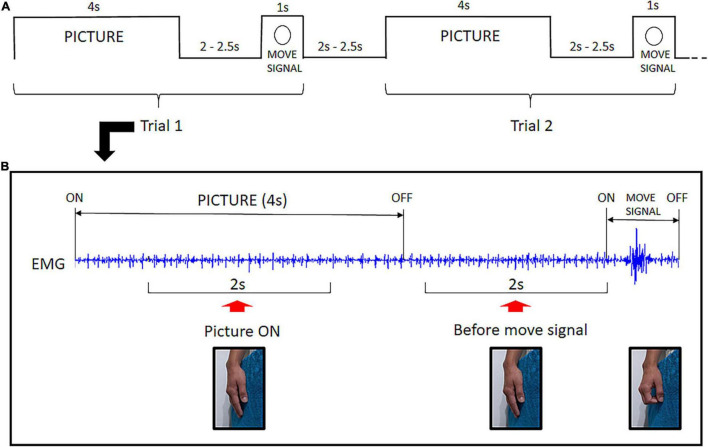
Experimental Design: **(A)** Example of the temporal structure of two consecutive trials. Each picture was presented for 4 s followed by an interval varying from 2.0 to 2.5 s. Then the move signal appeared (annulus) setting off the flexion of fingers. After a 2.0 to 2.5 s interval, a new trial began. **(B)** Raw EMG signal excerpted from a single trial illustrating the two time-windows for EMG analysis. The *Picture ON* context was investigated throughout a 2 s EMG segment during picture exposure. The *before move signal* context refers to a 2 s EMG segment preceding the task signal for fingers flexion. No overt hand movement is present before the move signal.

A fixation cross was displayed in the center of the screen throughout the experiment. Each trial began with the display of a picture on the screen. The picture was presented for 4 s, and the participant was instructed to look at it as long as it was displayed. Then, the picture turned off and, after an interval varying from 2.0 to 2.5 s, a small annulus appeared around the fixation cross which was the signal to execute a quick flexion of the fingers over the soft cloth. Then the participant returned the fingers to the initial relaxed position. Following another interval of 2.0 to 2.5 s, a new trial began with the display of the next picture of the same category (see Figure 2A).

After 20 trials, the block ended, and the participant rested their hand on their lap for a few minutes and waited for the instructions to start a new block.

Four blocks, two for each condition, were presented to each participant, totalizing 40 trials per condition. The condition starting the session was balanced between participants. The four possible orders of blocks were the following:

(i) set 1 (control) / set 1 (bonding) / set 2 (control) / set 2 (bonding);
(ii) set 2 (control) / set 2 (bonding) / set 1 (control) / set 1 (bonding);
(iii) set 1 (bonding) / set 1 (control) /set 2 (bonding) / set 2 (control);
(iv) set 2 (bonding) / set 2 (control) / set 1 (bonding) / set 1 (control).

### Data Analysis

The EMG signal was band-pass filtered from 20 to 250 Hz and segmented based on triggers recorded along with the signal. Evidence of caress-like motor pre-dispositions evoked by bonding pictures was investigated in two different contexts: *Picture ON* and *before move* signal. Preceding the signal to execute the fingers‘ flexion, there was a 2 s-period during which the picture had been turned off for all trials. This was selected as the *before move signal* time-window for EMG analysis. To investigate EMG modulation during picture exposure (*Picture ON*), we selected a 2 s-period, following the initial 1 s after picture ‘onset and ending before the final 1 s before its offset (Figure 2B).

All EMG segments were visually inspected by the experimenters in order to exclude those presenting artifacts or any evidence of voluntary muscle contraction. The EMG activity at rest is a signal of low amplitude, thus, for a reliable approach to calculate signal intensity, we applied Fast Fourier Transform (FFT) to the valid EMG segments. This transformation allowed the exclusion of 60 Hz-line noise (± 5 Hz) and its harmonic bands from the analysis. The FFT power spectra, representing signal intensity, were computed for each trial separately for the two windows of interest.

To further guarantee data integrity, we searched for spurious electrical activity by examining the average of trials within each block and excluding those with outlier spectral power values (above and below mean plus and minus 2.5 standard deviations). Excluded trials in any analysis were below 5%.

It is known that surface electromyography shows a great variation between individuals due to skin and fat thickness, among other factors (Farina et al., 2004). Thus, for statistical analysis, power values were normalized among the trials to vary from zero to one (min-max normalization), applying the formula (value–minimum value)/ (maximum value–minimum value).

We used two statistical approaches for testing our hypothesis. One approach relied on averaging all 40 trials, respectively, for the bonding and control conditions, for each participant (participant-level analysis). In the second approach, we collapsed the values from all participants for each bonding dyad and for the corresponding matching control, resulting in one mean value per picture (picture-level analysis). Picture-level analyses are widely used in studies of affective pictures (e.g., Bradley et al., 2001), to examine how consistently the individual exemplars in a given picture category (here, bonding vs. control) evoke a response. Thus, for this approach, we investigated if the response pattern evoked by the 26 bonding pictures was different from the responses evoked by the respective matched control picture. To avoid any possible effect due to picture repetition, this analysis used only data from the first picture exposure. The mean of the normalized values of the control and bonding conditions were calculated within the trials used for each approach.

For all analyses, each context (picture ON and before move signal) was investigated separately. To address the challenge of a smaller sample, the focus here was on conducting few, theory-driven, comparisons (bonding vs. control) rather than every possible comparison. Comparisons of the control and bonding conditions were performed through Wilcoxon matched-pairs tests, to account for the pilot sample size. For all analyses, *p*-values ≤ 0.05 were considered statistically significant.

## Results

**FIGURE 3:**
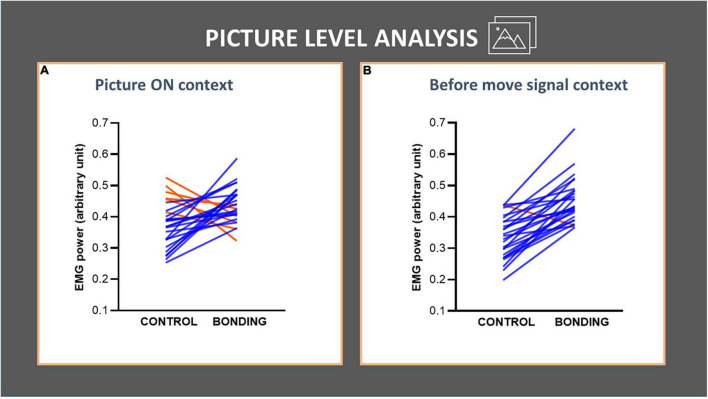
Picture-level analysis in each context **(A,B)** of the finger flexor muscle EMG activity. The lines joining control and bonding conditions represent normalized EMG power for each of the 26 dyads, averaged from all participants, i.e., each line represents a pair of control and its respective bonding picture. Blue lines represent data for dyads with increased values in bonding relative to the control conditions and red lines represent the opposite. **(A)** *Picture ON* context: observe that 20 out of 26 pairs showed an increase in EMG activity for the bonding condition relative to the matched control. **(B)** *Before move signal* context: observe that 25 out of 26 pairs showed an increase in EMG activity for the bonding condition relative to the matched control.

In the picture level analysis, we focused on the pairwise pictures approach. The pictures depicted 26 different dyads photographed in the same environment, either socially interacting or not interacting. We tested if the social interaction, being the salient difference between the two pictures of the same dyad, increased the background EMG activity of the finger flexor muscle by comparing the 26 matched pairs. For the *Picture ON* context, Wilcoxon matched-pairs tests revealed significantly higher EMG activity for the bonding condition compared to the matched control condition (*N* = 26, *Z* = 2.68, *p* = 0.007, effect size *r* = 0.37). In the *before move signal* context we also observed an increase in EMG activity for the bonding condition compared to the matched control condition (*N* = 26, *Z* = 4.28, *p* = 0.00002, effect size *r* = 0.59). Values in the bonding condition are higher than in the control one in 20 of the 26 pairs during the *picture ON* context (Figure 3A), and in 25 out of the 26 pairs during the *before move signal* context (Figure 3B and see Supplementary Table 1). These confirmed our prediction that the capture of bonding cues would impact the background EMG activity of the fingers’ flexor muscle, in the absence of any overt movement.

**FIGURE 4:**
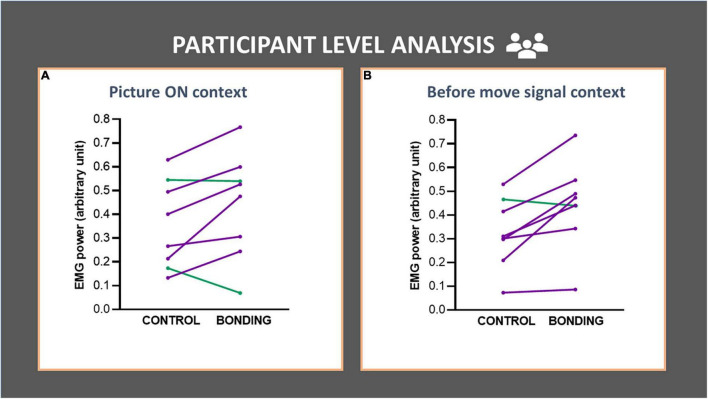
Participant-level analysis in each context **(A,B)** of the finger flexor muscle EMG activity. Each line represents data for a participant (averaged from all trials). EMG activity of the finger flexor muscle is depicted in the bonding and control conditions. **(A)** *Picture ON* context and **(B)** *before move signal* context. The lines joining control and bonding conditions represent normalized EMG power for each participant, averaged from all trials. Purple lines represent data for participants with increased values in bonding relative to the control conditions and green lines represent the opposite.

In the participant-level analysis we compared EMG activity in the bonding and control conditions across participants (*N* = 8). For the Picture ON context, we observed a higher EMG activity in the bonding condition than in the control condition (*Z* = 1.96, *p* = 0.050, effect size *r* = 0.49). This increment in EMG activity was also present in the before move signal context (*Z* = 2.24, *p* = 0.025, effect size *r* = 0.56). These results are depicted in Figures 4A,B (see Supplementary Table 2).

## Discussion

Finger flexion is an essential component of social grooming. In the present pilot study, we aimed to test the hypothesis that exposure to social interaction prompts modulation of the activity in the effector system, specifically the muscular system underlying social grooming. Dyads, child-child or child-adult, were photographed against the same background in two conditions: interacting (bonding) and non-interacting (control). The electromyographic activity of the participants’ fingers flexor muscle was the target of this preliminary investigation. Exposure to socially interacting dyads significantly and sustainably increased participant’s average EMG activity compared to exposure to non-interacting dyads. More importantly, when comparing each pair of matched pictures, the one depicting the interacting dyad consistently evoked higher EMG activity in comparison with the one depicting the same dyad not interacting. The sole change in attitudes of the dyads, from non-interacting to interacting, impacted EMG activity.

Our previous work (Campagnoli et al., 2015), examining the readiness potential at the cortical motor area, indicated a preset circuit linking the capture of bonding cues (from pictures) to motor preparation for grooming-like hand movement. In the present pilot study, by analyzing the electromyographic activity of the fingers’ flexor muscle, and having each dyad photographed in the same background in opposing attitudes–non-interacting and interacting, we further explored the visuo-motor affective interplay.

In the present preliminary results, we found modulation of background EMG activity of finger flexor during the passive exposure to bonding pictures. The increased EMG activity prompted by the visualization of interacting dyads is important and new. The EMG signal of fingers flexor represents the electrical activity generated in its muscle fibers in response to the activation provided by their exclusively innervating motor neurons in the spinal cord. Specific capture of visual bonding cues at the level of visual cortex was already highlighted in Papeo (2020). The increased EMG activity recorded during exposure to pictures of interacting dyads indicates that this processing reaches the spinal motor neurons responsible for driving the finger flexor muscle, adding to the hypothesized link between bonding cues and predisposition to grooming-like action, even during the passive viewing context.

Analyses of the *before move signal* context, when the picture is OFF and the participant is expecting the signal to perform the finger flexion task, revealed that previous exposure to an interacting dyad impacted on background EMG activity. An interpretation of this result is that motor preparation for a task performed by finger flexor muscle is being affected by the previous bonding cue in a sustained way. The enhanced EMG background activities might reflect a sustained leakage of central motor activity downstream, resulting in a slight but significant increase in firing of the respective motor neurons. Thus, the results from the analysis of both contexts (*Picture ON* and *before move signal*) support a lasting impact of visuo-motor association of bonding cues to predisposition to giving grooming.

The findings presented here inform a broader range of questions related to social cognition and behavior: Proximity and interpersonal social contact are important components of social connection, which we are constantly seeking and engaged in maintaining (Nelson and Geher, 2007; Dunbar, 2010; Gallace and Spence, 2010; Morrison, 2016). Social touch is crucial for survival and wellbeing at all stages of human life (Feldman and Eidelman, 2003; Brauer et al., 2016; Sehlstedt et al., 2016; Charpak et al., 2017; Cruciani et al., 2021).

Receiving social touch is unequivocally linked to social interaction. The physiology of sensory fibers tuned to receiving social touch has been largely explored during the last two decades. A specialized system, the C-tactile pleasant touch pathway, underlying the processing of receiving social pleasant touch, was thoroughly described (Löken et al., 2009; Morrison et al., 2010; Gazzola et al., 2012; Lloyd et al., 2013; Ackerley et al., 2014). The countless benefits of receiving social touch (e.g., Feldman et al., 2010; Field, 2010, 2019; Jakubiak and Feeney, 2017) and the pitfalls of not receiving it (Field, 2014; von Mohr et al., 2021) have been extensively investigated.

Much less attention has been given to the vitally important motivational system for giving touch, for which we envisage to contribute with the present pilot study. Maturana and Verden-Zöller (2008) proposed that human hands are caressing organs. The tactile exploration of surfaces’ pleasantness was shown to involve vibration-sensitive Pacinian Corpuscles in hand palm, as well as proprioceptive afferents (Klöcker et al., 2013). Gentsch et al. (2015) showed that actively touching others’ skin elicits sensory and haptic pleasure in the giver, possibly involving the same receptors described by Klöcker et al. (2013).

Although the “toucher side” is still underexplored (but see Lo et al., 2021), there are many reports of health benefits for the “giver” of pleasant touch. Aureli and Yates (2010) and Kajokaite et al. (2020) have reported, in non-human primates, significant stress reduction for the grooming giver. Indeed, Shutt et al. (2007) had shown previously that it was giving grooming, rather than receiving grooming, which was linked with physiological stress reduction. In his pioneering work, Harlow (1959) described how the availability of a “doll” covered with soft cloth mitigated the negative effects of isolation in young Rhesus monkeys. Although his work is frequently cited in the context of receiving social touch, it should be noted that the young monkey in this study attained contact comfort by being the agent of touch. Human benefits of giving grooming were examined in a review of human-animal interactions (Beetz et al., 2012). These authors found a notable number of well-controlled studies showing that giving grooming to an animal evokes positive psychological and psychophysiological effects in humans. Tai et al. (2011) showed in human adults that touching a teddy-bear mitigates the effects of social exclusion. Giving regular massage to one’s own infant was shown to bring benefits to parents (Cullen et al., 2000; Onozawa et al., 2001).

The importance of giving and receiving social touch has been highlighted in the current COVID-19 pandemic for which social isolation, the most effective protective strategy to stop virus spreading, leads to hazardous effects of touch deprivation (Bzdok and Dunbar, 2020; Dezecache et al., 2020; von Mohr et al., 2021). Dogs’ adoption boomed during the current pandemic (Morgan et al., 2020), partially compensating the need for social contact by providing the possibility of giving grooming (Beetz et al., 2012).

## Limitations and Conclusion

The small and female-biased sample is an important limitation of the present work. It could be argued that the results may be specific to women. Still, the findings were very consistent among the sample, with all (including the sole male participant) but one observer responding to bonding cues with heightened EMG activity during the before move signal context and all (again including the sole male participant) but two observers, showing the same pattern during the *Picture ON* period. Future work will aim to replicate these preliminary findings in a larger sample, prevented in the present case by the global pandemic. In addition, recording a muscle not specifically related to grooming-like movements may provide additional control and assist in establishing specificity.

In this pilot study, we examined the effects of viewing social bonding cues on covert motor output, implicated in the predisposition for social grooming in a small sample of participants. The electromyographic activity of the finger flexor, an important muscle when performing social grooming, was increased due to the exposure of interacting dyads pictures while participants were not making any overt movement. These preliminary data suggest an implicit visuo-motor connection in which social interaction cues evoke intrinsic dispositions toward affectionate social behavior.

## Data Availability Statement

The raw data supporting the conclusions of this article will be made available by the authors, without undue reservation.

## Ethics Statement

The studies involving human participants were reviewed and approved by the Ethics Review Board of the Institute of Psychiatry of the Federal University of Rio de Janeiro. The patients/participants provided their written informed consent to participate in this study.

## Author Contributions

EV, JO, VR-R, ID, RC, SG, AK, OG, CV, CF, and MP: conceptualization. EV, JO, VR-R, ID, RC, SG, OG, CF, and MC: methodology. JO: software. EV, JO, and VR-R: formal analysis and supervision. EV, JO, VR-R, OG, and CF: investigation. EV, JO, VR-R, and OG: writing. EV, JO, VR-R, ID, GS, RC, SG, AK, OG, CV, and MP: writing - review and editing. All authors contributed to the article and approved the submitted version.

## Conflict of Interest

The authors declare that the research was conducted in the absence of any commercial or financial relationships that could be construed as a potential conflict of interest.

## Publisher’s Note

All claims expressed in this article are solely those of the authors and do not necessarily represent those of their affiliated organizations, or those of the publisher, the editors and the reviewers. Any product that may be evaluated in this article, or claim that may be made by its manufacturer, is not guaranteed or endorsed by the publisher.
