# Supplementary material for: Visuo-Motor Affective Interplay: Bonding Scenes Promote Implicit Motor Pre-dispositions Associated With Social Grooming–A Pilot Study
Source: Front Psychol. 2022 Apr 7;13:817699. doi: 10.3389/fpsyg.2022.817699 (PMC9022038; doi:10.3389/fpsyg.2022.817699)
Supplement: Supplementary file 1 [file Data_Sheet_1.PDF]

Table S1: Picture-level analysis. Normalized EMG power averaged from all participants (resulting in one mean value per picture) for each bonding dyad and for the corresponding matching control. Dyads are numbered 1 to 26. Median and median absolute deviation (M.A.D.) are shown.

PICTURE ON		
**Dyad**	**Control**	**Bonding**
1	0.2134	0.5978
2	0.4192	0.5285
3	0.4819	0.2296
4	0.1377	0.3906
5	0.3091	0.4623
6	0.3393	0.4911
7	0.3027	0.4344
8	0.4951	0.1367
9	0.2188	0.5102
10	0.4037	0.4358
11	0.4252	0.5228
12	0.2313	0.5505
13	0.4594	0.2594
14	0.3201	0.5406
15	0.4339	0.4325
16	0.1449	0.5536
17	0.3522	0.6491
18	0.1546	0.5525
19	0.4413	0.6075
20	0.2493	0.3972
21	0.5555	0.4663
22	0.1484	0.6531
23	0.2532	0.4999
24	0.5286	0.5422
25	0.1840	0.3553
26	0.3586	0.4736
Median	0.3297	0.4955
M.A.D.	0.1075	0.0589

BEFORE MOVE SIGNAL		
**Dyad**	**Control**	**Bonding**
1	0.1723	0.5352
2	0.2808	0.3697
3	0.5021	0.7047
4	0.1812	0.3536
5	0.3420	0.3605
6	0.3776	0.4662
7	0.2981	0.3293
8	0.3691	0.4420
9	0.1837	0.5237
10	0.3880	0.5661
11	0.3229	0.4667
12	0.3478	0.4675
13	0.4640	0.4033
14	0.2354	0.4543
15	0.4407	0.4503
16	0.1100	0.3687
17	0.4333	0.5730
18	0.2188	0.5116
19	0.3942	0.5552
20	0.2171	0.4182
21	0.3921	0.4309
22	0.3647	0.5263
23	0.2857	0.4741
24	0.3741	0.5542
25	0.3262	0.7680
26	0.4075	0.4413
Median	0.3449	0.4665
M.A.D.	0.0609	0.0615

Table S2: Participant-level analysis. Values of normalized EMG power (average of all 40 trials) for each participant in the control and bonding conditions. Median and median absolute deviation (M.A.D.) are shown.

PICTURE ON		
**Participant**	**Control**	**Bonding**
P1	0.6294	0.7670
P2	0.4952	0.5996
P3	0.5447	0.5399
P4	0.2137	0.4757
P5	0.4011	0.5262
P6	0.1328	0.2443
P7	0.2661	0.3062
P8	0.1735	0.0692
Median	0.3336	0.5009
M.A.D.	0.1609	0.1467

BEFORE MOVE SIGNAL		
**Participant**	**Control**	**Bonding**
P1	0.5295	0.7359
P2	0.4152	0.5473
P3	0.4640	0.4375
P4	0.2088	0.4733
P5	0.3104	0.4401
P6	0.2977	0.4900
P7	0.3010	0.3427
P8	0.0733	0.0865
Median	0.3057	0.4567
M.A.D.	0.1032	0.0619

Note: P6 is the male participant
